# Supplementary figures and images for: Compromised base excision repair pathway in Mycobacterium tuberculosis imparts superior adaptability in the host
Source: PLoS Pathog. 2021 Mar 19;17(3):e1009452. doi: 10.1371/journal.ppat.1009452 (PMC8011731; doi:10.1371/journal.ppat.1009452)

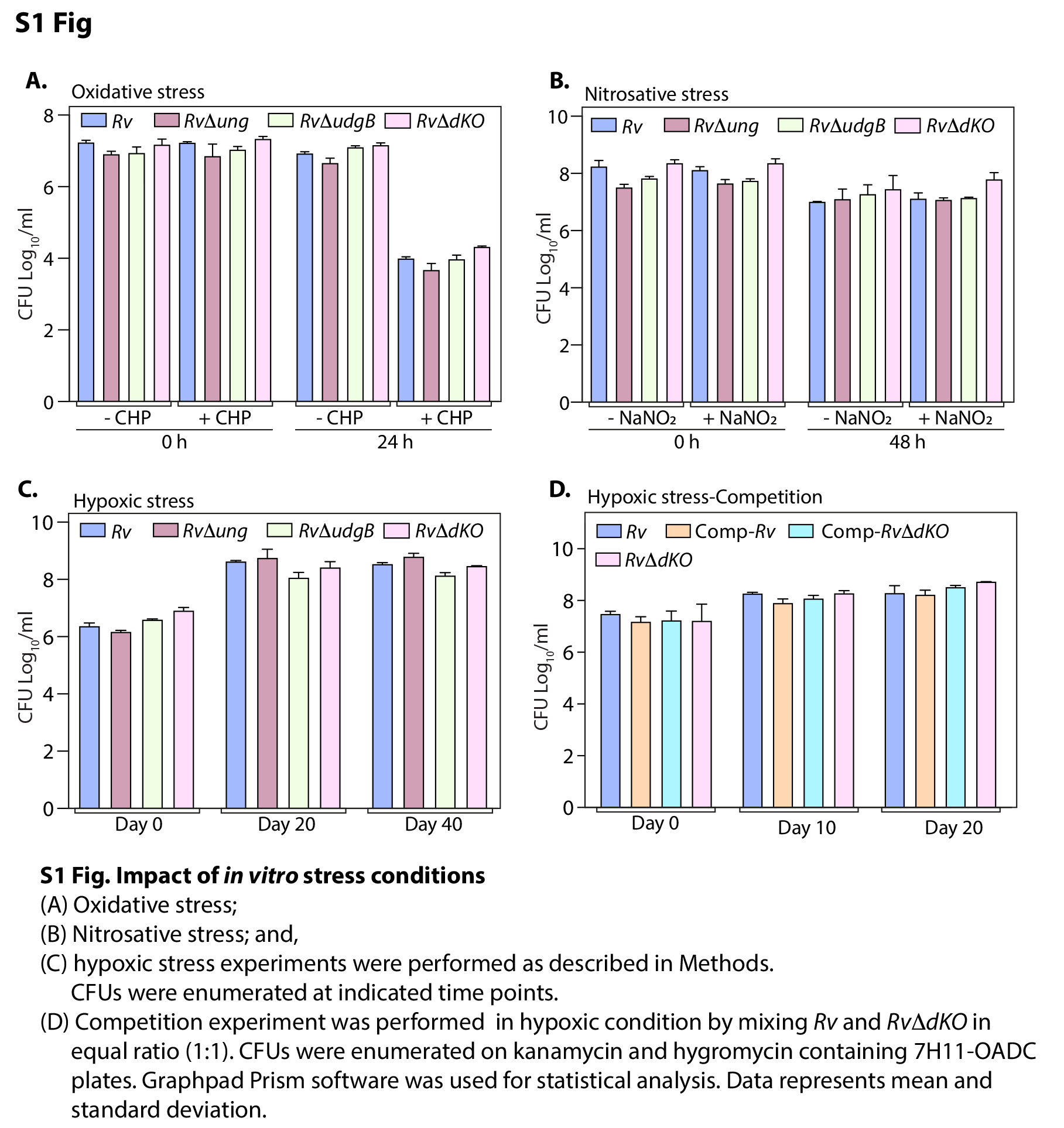

Supplement: S1 Fig — (A) Oxidative stress; (B) Nitrosative stress; and (C) hypoxic stress experiments were performed as described in Methods. CFUs were enumerated at indicated time points. (D) Competition experiment was performed in hypoxic condition by mixing Rv and RvΔdKO in equal ratio (1,1). CFUs were enumerated on kanamycin and hygromycin containing 7H11-OADC plates. Graphpad Prism software was used for statistical analysis. Data represents mean and standard deviation. (TIF) [file ppat.1009452.s001.tif]

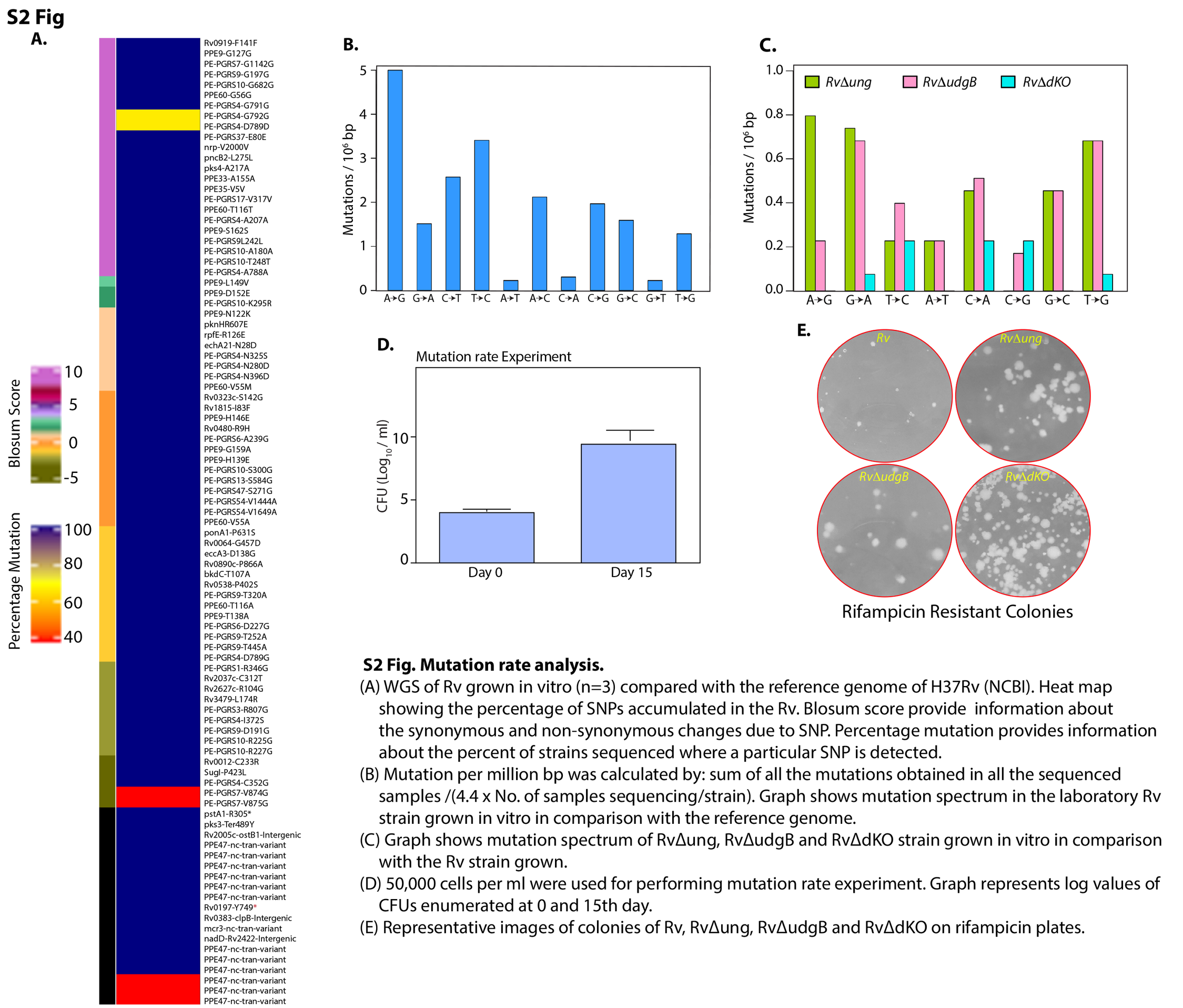

Supplement: S2 Fig — (A). WGS of Rv grown in vitro (n = 3) compared with the reference genome of H37Rv (NCBI). Heat map showing the percentage of SNPs accumulated in the Rv. Blosum score provide information about the synonymous and non-synonymous changes due to SNP. Percentage mutation provides information about the percent of strains sequenced where a particular SNP is detected. (B) Mutation per million bp was calculated by: sum of all the mutations obtained for a in all the sequenced samples /(4.4 x No. of samples sequencing/strain). Graph shows mutation spectrum in the laboratory Rv strain grown in vitro in comparison with the reference genome. (C) Graph shows mutation spectrum of RvΔung, RvΔudgB and RvΔdKO and strains grown in vitro in comparison with Rv strain. (D) 50,000 cells per ml were used for performing mutation rate experiment. Graph represents log values of CFUs enumerated at 0 and 15th day. (E) Representative images of colonies of Rv, RvΔung, RvΔudgB and RvΔdKO on rifampicin plates. (TIF) [file ppat.1009452.s002.tif]

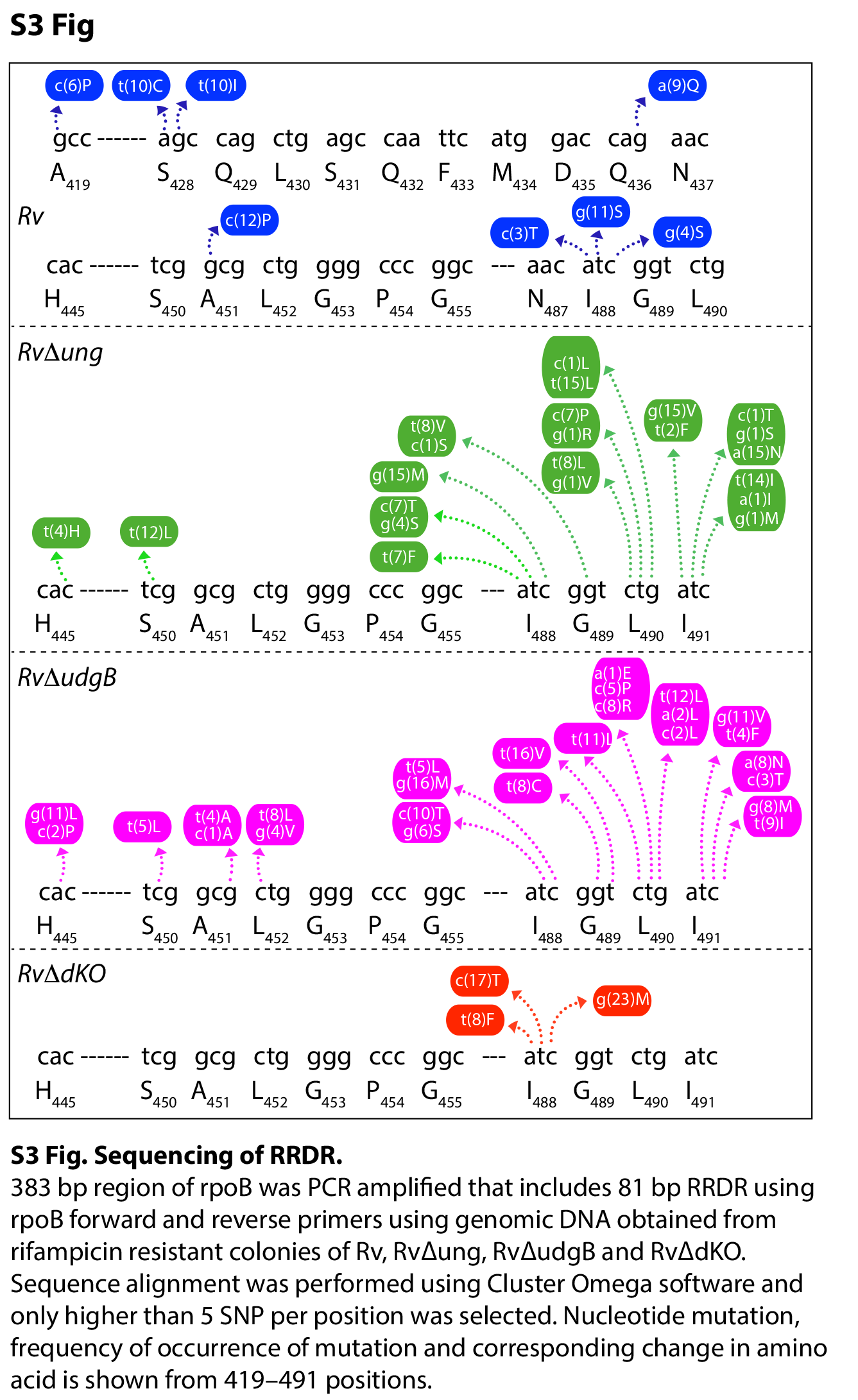

Supplement: S3 Fig — 383 bp region of rpoB was PCR amplified that includes 81 bp RRDR using rpoB forward and reverse primers using genomic DNA obtained from rifampicin resistant colonies of Rv, RvΔung, RvΔudgB and RvΔdKO. Sequence alignment was performed using Cluster Omega software and only higher than 5 SNP per position was selected. Nucleotide mutation, frequency of occurrence of mutation and corresponding change in amino acid is shown from 419–491 positions. (TIF) [file ppat.1009452.s003.tif]

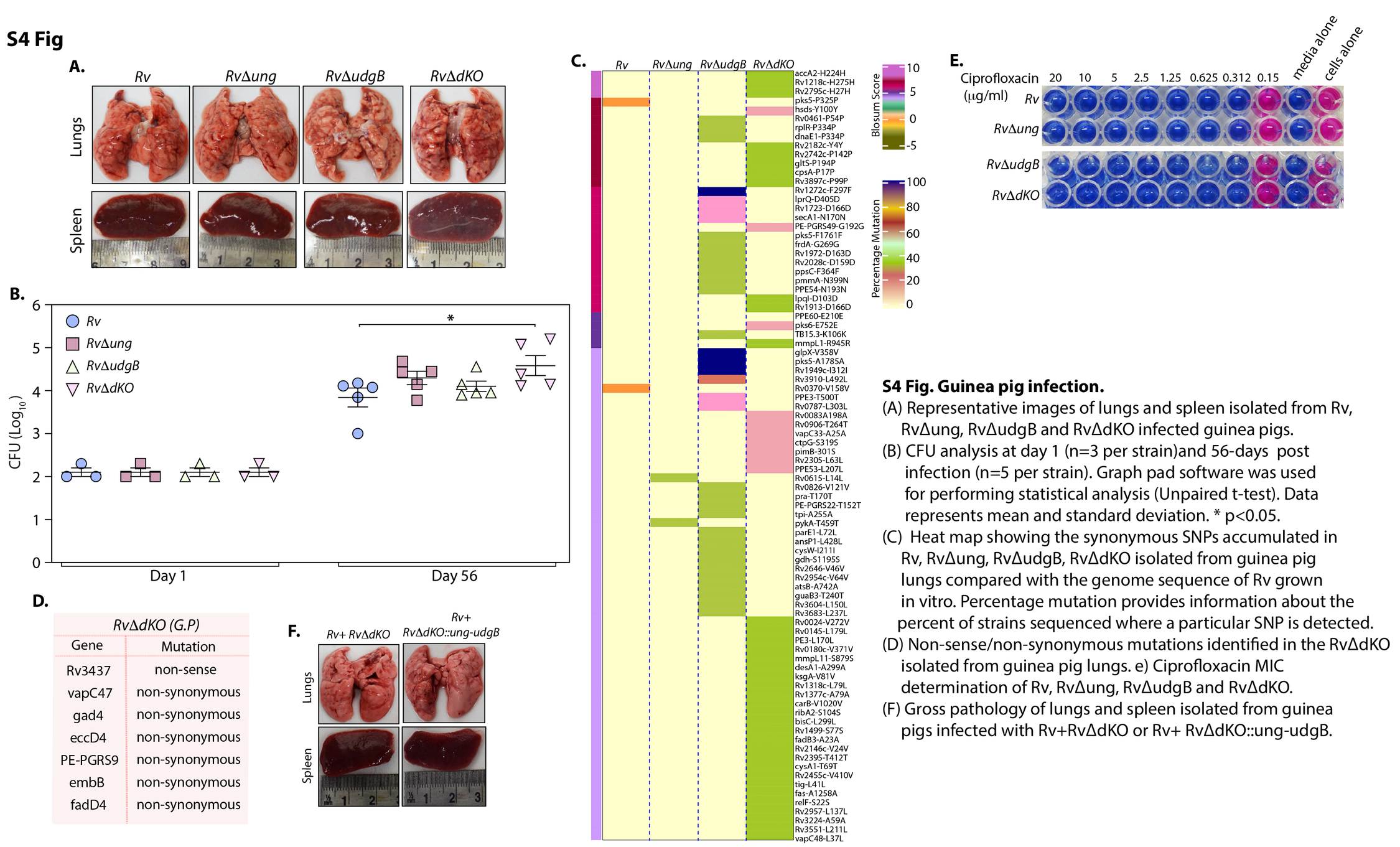

Supplement: S4 Fig — (A) Representative images of lungs and spleen isolated from Rv, RvΔung, RvΔudgB and RvΔdKO infected guinea pigs. (B) CFU analysis at day 1 (n = 3 per strain) and 56 days post infection (n = 5 per strain). Graph pad software was used for performing statistical analysis (Unparied t-test). Data represents mean and standard deviation. * p<0.05. (C) Heat map showing the synonymous SNPs accumulated in Rv, RvΔung, RvΔudgB, RvΔdKO isolated from guinea pig lungs compared with the sequence of Rv grown in vitro. Percentage mutation provides information about the percent of strains sequenced where a particular SNP is detected. (D) Non-sense/non-synonymous mutations identified in the RvΔdKO isolated from guinea pig lungs. (E) Ciprofloxacin MIC determination of Rv, RvΔung, RvΔudgB and RvΔdKO. f) Gross pathology of lungs and spleen isolated from guinea pigs infected with Rv +RvΔdKO or Rv +RvΔdKO::ung-udgB. (TIF) [file ppat.1009452.s004.tif]

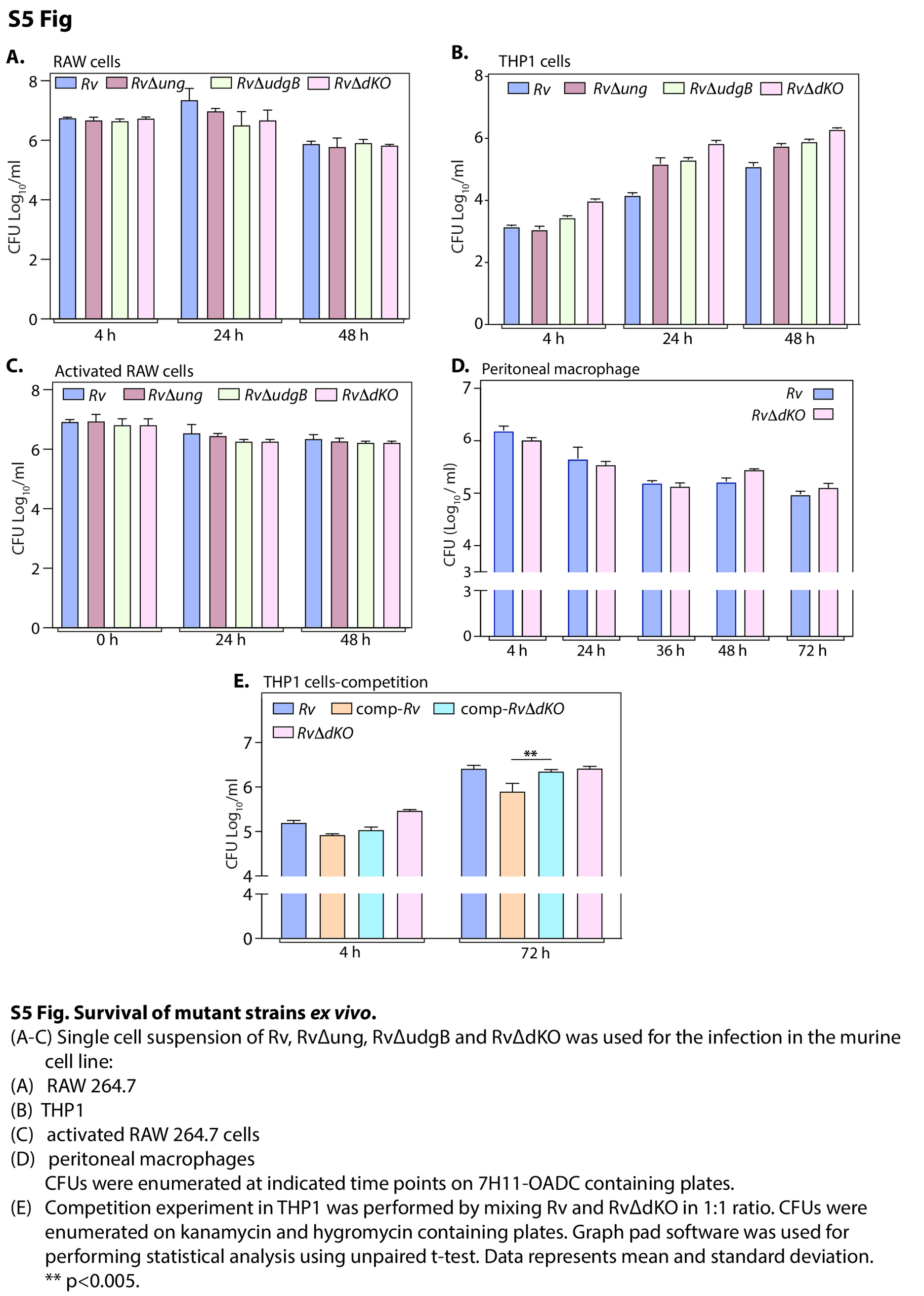

Supplement: S5 Fig — (A-c) Single cell suspension of Rv, RvΔung, RvΔudgB and RvΔdKO was used for the infection in the murine cell line (A). RAW 264.7; (B) THP1; (C) activated RAW 264.7 cells and, (D) peritoneal macrophages. CFUs were enumerated at indicated time points on 7H11-OADC containing plates. (E) Competition experiment in THP1 was performed by mixing Rv and RvΔdKO in 1:1 ratio. CFUs were enumerated on kanamycin and hygromycin containing plates. Graph pad software was used for performing statistical analysis using unpaired t-test. Data represents mean and standard deviation. ** p<0.005. (TIF) [file ppat.1009452.s005.tif]
