# Supplementary material for: Compromised base excision repair pathway in Mycobacterium tuberculosis imparts superior adaptability in the host
Source: PLoS Pathog. 2021 Mar 19;17(3):e1009452. doi: 10.1371/journal.ppat.1009452 (PMC8011731; doi:10.1371/journal.ppat.1009452)
Supplement: S1 Text — (DOCX) [file ppat.1009452.s006.docx]

**S1 Text**

***Growth kinetics and survival under in vitro stress conditions.***

*Rv,RvΔung, RvΔudgB* and *RvΔdKO* grown either in 7H9-ADC medium. For oxidative stress, strains inoculated at A_600_ ~0.2 were subjected to 50 μM cumene hydroperoxide (CHP) for 24 h. Nitrosative stress was generated by growing the cells in the presence of 3mM sodium nitrite in 7H9 medium (pH-5.5) for 48 h. For hypoxia stress, strains were grown to A_600_~ 0.1 in 7H9-ADC medium containing 1.5 µg/ml methylene blue. Hypoxia was established in 2 ml cryovials and CFUs were enumerated at day 0, 20 and 40 days. Competition experiment was performed in hypoxic condition by mixing *Rv* and *RvΔdKO* in 1:1.
