# Supplementary material for: Compromised base excision repair pathway in Mycobacterium tuberculosis imparts superior adaptability in the host
Source: PLoS Pathog. 2021 Mar 19;17(3):e1009452. doi: 10.1371/journal.ppat.1009452 (PMC8011731; doi:10.1371/journal.ppat.1009452)
Supplement: S2 Text — (DOCX) [file ppat.1009452.s007.docx]

**S2 Text**

***Mutation rate analysis***

Mutation rate analysis is performed as described in the Methods.
