# Supplementary material for: Compromised base excision repair pathway in Mycobacterium tuberculosis imparts superior adaptability in the host
Source: PLoS Pathog. 2021 Mar 19;17(3):e1009452. doi: 10.1371/journal.ppat.1009452 (PMC8011731; doi:10.1371/journal.ppat.1009452)
Supplement: S3 Text — (DOCX) [file ppat.1009452.s008.docx]

**S3 Text**

***Sequencing of RRDR.***

Rifampicin resistant colonies were grown upto A_600_ ~0.8 and cells were resuspended in 200 μl of TE and equal volume of chloroform was added. Contents were heated in dry bath at 90^o^C for 15 min followed by centrifugation at 13000 rpm for 45 min at 4^o^C and the aqueous layer was collected. After confirming the genomic DNA integrity by gel, the aqueous layer was used as the template for PCR to amplify RRDR. Amplicons were ethanol precipitated, resuspended in 1X TE and 500 ng was sent for sequencing (Macrogen).
