# Supplementary material for: Compromised base excision repair pathway in Mycobacterium tuberculosis imparts superior adaptability in the host
Source: PLoS Pathog. 2021 Mar 19;17(3):e1009452. doi: 10.1371/journal.ppat.1009452 (PMC8011731; doi:10.1371/journal.ppat.1009452)
Supplement: S5 Text — (DOCX) [file ppat.1009452.s010.docx]

**S5 Text**

***Survival of mutants ex vivo.***

*Ex vivo* infection experiments were performed as described previously [1]. All the above experiments were performed in two biological independent experiments and each time in triplicates. Statistical analysis (Unpaired t-test) was performed using Graphpad Prism. Data represents mean and SD. **p<0.005.

Reference

1. Lochab S, Singh Y, Sengupta S, Nandicoori VK. Mycobacterium tuberculosis exploits host ATM kinase for survival advantage through SecA2 secretome. Elife. 2020;9. doi: 10.7554/eLife.51466. PubMed PMID: 32223892; PubMed Central PMCID: PMCPMC7162654.
