# Supplementary material for: Compromised base excision repair pathway in Mycobacterium tuberculosis imparts superior adaptability in the host
Source: PLoS Pathog. 2021 Mar 19;17(3):e1009452. doi: 10.1371/journal.ppat.1009452 (PMC8011731; doi:10.1371/journal.ppat.1009452)
Supplement: S2 Table — (DOCX) [file ppat.1009452.s012.docx]

**Compromised base excision repair pathway in *Mycobacterium tuberculosis* imparts superior adaptability in the host**

Saba Naz^1,4^, Shruti Dabral^3^, Sathya Narayanan Nagarajan^1,5^, Divya Arora^1,6^, Lakshya Veer Singh^3^, Pradeep Kumar^2,7^, Yogendra Singh^4^, Dhiraj Kumar^3^, Umesh Varshney*^2^ and Vinay Kumar Nandicoori*^1^

**Supplementary Material**

***Sequencing of RRDR.***

Rifampicin resistant colonies were grown upto A_600_ ~0.8 and cells were resuspended in 200 μl of TE and equal volume of chloroform was added. Contents were heated in dry bath at 90^o^C for 15 min followed by centrifugation at 13000 rpm for 45 min at 4^o^C and the aqueous layer was collected. After confirming the genomic DNA integrity by gel, the aqueous layer was used as the template for PCR to amplify RRDR. Amplicons were ethanol precipitated, resuspended in 1X TE and 500 ng was sent for sequencing (Macrogen).

***Growth kinetics and survival under in vitro stress conditions.***

*Rv,RvΔung, RvΔudgB* and *RvΔdKO* grown either in 7H9-ADC medium. For oxidative stress, strains inoculated at A_600_ ~0.2 were subjected to 50 μM cumene hydroperoxide (CHP) for 24 h. Nitrosative stress was generated by growing the cells in the presence of 3mM sodium nitrite in 7H9 medium (pH-5.5) for 48 h. For hypoxia stress, strains were grown to A_600_~ 0.1 in 7H9-ADC medium containing 1.5 µg/ml methylene blue. Hypoxia was established in 2 ml cryovials and CFUs were enumerated at day 0, 20 and 40 days. Competition experiment was performed in hypoxic condition by mixing *Rv* and *RvΔdKO* in 1:1.

***Survival of mutants ex vivo.***

*Ex vivo* infection experiments were performed as described previously (1). All the above experiments were performed in two biological independent experiments and each time in triplicates. Statistical analysis (Unpaired t-test) was performed using Graphpad Prism. Data represents mean and SD. **p<0.005.

**Supplementary Figures.**

***S1 Fig. Impact of in vitro stress conditions.***

***S2. Fig. Mutation rate analysis***

***S3 Fig. Sequencing of RRDR***

***S4 Fig. Guinea pig infection***

***S5 Fig. Survival of mutant strains ex vivo.***

**Supplementary Tables**

***S1 Table: Comparison of Rv in vitro with reference Rv (NCBI).***

**Table given in the Supplementary excel file.**

***S2 Table: Mutation Spectrum of Rv in vitro.***

***S3 Table: Comparison of Rv in vitro with Rv****Δ****ung in vitro, Rv****Δ****udgB in vitro and Rv****Δ****dKO in vitro.***

**Table given in the Supplementary excel file.**

***S4 Table: Mutation spectrum of Rv****Δ****ung in vitro, Rv****Δ****udgB in vitro and Rv****Δ****dKO in vitro.***

***S5 Table: Comparison of Rv in vitro with Rv (G.P) isolated from guinea pig lungs.***

**Table given in the Supplementary excel file.**

***S6 Table: Mutation Spectrum of Rv (GP).***

***S7 Table: Comparison of Rv in vitro with Rv (GP), RvΔung (GP), RvΔudgB (GP) and RvΔdKO (GP).***

**Table given in the Supplementary excel file.**

***S8 Table: Mutation spectrum of Rv(GP), Rv****Δ****ung(GP), Rv****Δ****udgB(GP) and Rv****Δ****dKO(GP).***

***S9 Table: Comparison of Rv****Δ****dKO in vitro with Rv****Δ****dKO ciprofloxacin resistant (CR) strains.***

**Table given in the Supplementary excel file.**

***S10 Table: Mutation spectrum of Rv****Δ****dKO in vitro and Rv****Δ****dKO ciprofloxacin resistant strains.***

***S11 Table: List of DNA oligomers used in the study.***

***S12 Table. Source Data File.***

**Table given in the Supplementary excel file.**

***S2 Table: Mutation Spectrum of Rv in vitro. Mutation Spectrum of Rv laboratory strain H37Rv***

| **Table S2: Mutation Spectrum of *Rv* in vitro** | | | | | |
| --- | --- | --- | --- | --- | --- |
|  |  |  |  |  | **Mutation per million bp** |
| **Mutation** | ***Rv_*1** | ***Rv_*2** | ***Rv_*3** | **Sum** | **(*Rv*)** |
| A_G | 22 | 22 | 22 | 66 | 5 |
| G_A | 7 | 7 | 6 | 20 | 1.515151515 |
| T_C | 15 | 15 | 15 | 45 | 3.409090909 |
| C_T | 11 | 12 | 11 | 34 | 2.575757576 |
| T_G | 5 | 7 | 5 | 17 | 1.287878788 |
| A_C | 9 | 10 | 9 | 28 | 2.121212121 |
| A_T | 1 | 1 | 1 | 3 | 0.227272727 |
| C_A | 1 | 2 | 1 | 4 | 0.303030303 |
| C_G | 9 | 9 | 8 | 26 | 1.96969697 |
| G_C | 7 | 7 | 7 | 21 | 1.590909091 |
| G_T | 1 | 1 | 1 | 3 | 0.227272727 |

***S4 Table: Mutation spectrum of RvΔung in vitro, RvΔudgB in vitro and RvΔdKO in vitro. Mutation spectrum of RvΔung, RvΔudgB and RvΔdKO grown in vitro.***

| **Table S4: Mutation spectrum of *RvΔung in vitro,RvΔudgB in vitro* and *RvΔdKO in vitro*** | | | | | | | |
| --- | --- | --- | --- | --- | --- | --- | --- |
|  |  |  |  | **Mutation per million bp** | | | |
| **Mutation** | ***RvΔung***  **sum** | ***RvΔdKO***  **sum** | ***RvΔudgB***  **sum** | ***RvΔung*** | ***RvΔudgB*** | | ***RvΔdKO*** |
| A_G | 14 | NA | 4 | 0.795454545 | 0.227272727 | | NA |
| A_T | 4 | NA | 4 | 0.227272727 | 0.227272727 | | NA |
| C_A | 8 | 3 | 9 | 0.454545455 | 0.511363636 | | 0.227272727 |
| C_G | NA | 3 | 3 | NA | 0.170454545 | | 0.227272727 |
| G_A | 13 | 1 | 12 | 0.738636364 | 0.681818182 | | 0.075757576 |
| G_C | 8 | NA | 8 | 0.454545455 | 0.454545455 | | NA |
| T_C | 4 | 3 | 7 | 0.227272727 | 0.397727273 | | 0.227272727 |
| T_G | 12 | 1 | 12 | 0.681818182 | 0.681818182 | | 0.075757576 |
|  |  |  |  |  | |  |  |

***S6 Table. Mutation Spectrum of Rv (GP).***

| **Table S6: Mutation Spectrum of *Rv* (GP)** | | | | | | | | | | | | | |
| --- | --- | --- | --- | --- | --- | --- | --- | --- | --- | --- | --- | --- | --- |
|  |  |  |  |  |  |  |  |  |  |  |  |  | **Mutation per million bp** |
| **Mutation** | ***Rv* (GP) 1** | ***Rv* (GP) 2** | ***Rv* (GP) 3** | ***Rv* (GP) 4** | ***Rv* (GP) 5** | ***Rv* (GP) 6** | ***Rv* (GP) 7** | ***Rv* (GP) 8** | ***Rv* (GP) 9** | ***Rv* (GP) 10** | ***Rv* (GP) 11** | **sum** | **(*Rv* GP)** |
| C_A | 1 | 1 | NA | 1 | NA | 1 | NA | NA | 1 | 1 | NA | 6 | 0.123966942 |
| C_G | 1 | NA | NA | 1 | NA | 1 | NA | NA | NA | 1 | NA | 4 | 0.082644628 |
| C_T | 1 | 1 | NA | 1 | 1 | 1 | NA | NA | 1 | 1 | NA | 7 | 0.144628099 |
| G_T | NA | NA | 1 | NA | NA | NA | NA | NA | NA | NA | 1 | 2 | 0.041322314 |
| T_G | NA | NA | 1 | NA | 1 | NA | NA | NA | 1 | NA | NA | 3 | 0.061983471 |
| G_A | NA | NA | NA | NA | 1 | NA | NA | NA | NA | NA | NA | 1 | 0.020661157 |
| A_G | NA | NA | NA | NA | NA | NA | 1 | 1 | NA | NA | NA | 2 | 0.041322314 |
| G_C | NA | NA | NA | NA | NA | NA | 1 | NA | NA | NA | NA | 1 | 0.020661157 |

| **Table S8: Mutation spectrum of *Rv* (GP)*, RvΔung* (GP)*, RvΔudgB* (GP) *and RvΔdKO* (GP)** | | | | | | | | |
| --- | --- | --- | --- | --- | --- | --- | --- | --- |
|  |  |  |  |  | **Mutation per million bp** | | | |
| **Mutation** | ***RvΔdKO* (GP) sum** | ***Rv* (GP) sum** | ***RvΔudgB* (GP)**  **sum** | ***RvΔung*(GP) sum** | ***Rv* (GP)** | ***RvΔung* (GP)** | ***RvΔudgB* (GP)** | ***RvΔdKO* (GP)** |
| A_G | NA | 2 | NA | NA | 0.041322314 | NA | NA | NA |
| C_A | 8 | 6 | 9 | 8 | 0.123966942 | 0.227272727 | 0.255681818 | 0.202020202 |
| C_G | 9 | 4 | 8 | NA | 0.082644628 | NA | 0.227272727 | 0.227272727 |
| C_T | 65 | 7 | 72 | 5 | 0.144628099 | 0.142045455 | 2.045454545 | 1.641414141 |
| G_A | 71 | 1 | 86 | NA | 0.020661157 | NA | 2.443181818 | 1.792929293 |
| G_C | NA | 1 | NA | NA | 0.020661157 | NA | NA | NA |
| G_T | NA | 2 | NA | NA | 0.041322314 | NA | NA | NA |
| T_C | 8 | NA | 8 | 1 | NA | 0.028409091 | 0.227272727 | 0.202020202 |
| T_G | 3 | 3 | 2 | 6 | 0.061983471 | 0.170454545 | 0.056818182 | 0.075757576 |

***S8 Table. Mutation spectrum of Rv (GP), RvΔung (GP), RvΔudgB (GP) and RvΔdKO(GP).***

***S10 Table. Mutation spectrum of RvΔdKO in vitro and RvΔdKO ciprofloxacin resistant strains.***

| **Table S10: Mutation spectrum of *RvΔdKO in vitro* and *RvΔdKO* CR** | | | | |
| --- | --- | --- | --- | --- |
|  |  |  | **Mutation per million bp** | |
| **Mutation** | ***RvΔdKO*** | ***RvΔdKO* CR** | ***RvΔdKO*** | ***RvΔdKO CR*** |
| A_G | NA | 13 | NA | 0.227272727 |
| C_A | NA | 1 | NA | 0.017482517 |
| C_T | NA | 63 | NA | 1.101398601 |
| G_A | 1 | 46 | 0.025252525 | 0.804195804 |
| T_G | 1 | 6 | 0.025252525 | 0.104895105 |

***S11 Table. List of DNA oligomers used in the study.***

| S.NO. | **Table 11: List of oligonucleiotides** | |
| --- | --- | --- |
| 1 | *rpoB* forward | 5’ CGACCACTTCGGCAACCG 3’ |
| 2 | *rpoB* reverse | 5’ CGATCAGACCGATGTTGG 3' |
| 3 | 5' flank forward primer of *ung* | 5*'* AAGCTTCGAGTCGCCGCATCCTCC 3*'* |
| 4 | 5' flank reverse primer of *ung* | 5*'* ACTAGTCGCAATACCGCGCCAGAG 3*'* |
| 5 | 3' flank forward primer of *ung* | 5' GGTACCGCTGGTGACAGATAGTCA 3*'* |
| 6 | 3' flank reverse primer of *ung* | 5*'* TCTAGAGCGCAACACATTCGATCC 3*'* |
| 7 | *Ung* knockout screening forward primer | 5’ CACTCACTTGCAACGGAGTCC 3’ |
| 8 | *Ung* knockout screening reverse primer | 5’ CAGCACCGAGACGATAGTTCC 3' |
| 9 | 5' flank forward primer of *udgB* | 5' TTTTTTTTCCACAAAGTGGTGATGCCGCCAACCG 3' |
| 10 | 5' flank reverse primer of *udgB* | 5' TTTTTTTTCCATTTCTTGGTTACGCCCGGCACACGCTGATC 3' |
| 11 | 3' flank forward primer of *udgB* | 5' TTTTTTTTCCACAGAGTGGGATTGAGTGACGTGAAGAC 3' |
| 12 | 3' flank reverse primer of *udgB* | 5' TTTTTTTTCCACCTTGTGGGTAGTCGTCACCGGCCGCC 3' |
| 13 | *UdgB* knockout screening forward primer | 5’ GATACAGCGCGGTGACGGTC 3’ |
| 14 | *UdgB* knockout screening reverse primer | 5’ ATGCACGATTCGTTCGAACTC 3’ |
| 15 | GU9 oligonuclieotide | 5'CTCAAGTGUAGGCATGCTTTTGCATGCCTGCACTTGA 3' |
| 16 | SSU9 oligonuclieotide | 5'CTCAAGTGUAGGCATGCAAGAGCT 3' |
| 17 | *Ung* gene forward primer with NdeI | 5’CACCCATATGGCCGCACGGCCGTTGAGT 3’ |
| 18 | *Ung* gene reverse primer with HindIII | 5’GATCAAGCTTCAGGGCAACCGCCAATCGAT 3’ |
| 19 | *UdgB* gene forward primer with NdeI | 5’cacccatatgaatatcgcggctgaatc 3’ |
| 20 | *UdgB* gene reverse primer with HindIII | 5’AGCTAAGCTTACTCAATCCCGGCCAGCTTC 3’ |

**References**

1. Lochab S, Singh Y, Sengupta S, Nandicoori VK. Mycobacterium tuberculosis exploits host ATM kinase for survival advantage through SecA2 secretome. Elife. 2020;9.
