# Supplementary material for: Compromised base excision repair pathway in Mycobacterium tuberculosis imparts superior adaptability in the host
Source: PLoS Pathog. 2021 Mar 19;17(3):e1009452. doi: 10.1371/journal.ppat.1009452 (PMC8011731; doi:10.1371/journal.ppat.1009452)
Supplement: S4 Table — (DOCX) [file ppat.1009452.s014.docx]

**S4 Table: Mutation spectrum of *RvΔung in vitro, RvΔudgB in vitro* and *RvΔdKO in vitro. Mu*tation spectrum of *RvΔung, RvΔudgB* and *RvΔdKO* grown *in vitro.***

| **Table S4: Mutation spectrum of *RvΔung in vitro,RvΔudgB in vitro* and *RvΔdKO in vitro*** | | | | | | | |
| --- | --- | --- | --- | --- | --- | --- | --- |
|  |  |  |  | **Mutation per million bp** | | | |
| **Mutation** | ***RvΔung***  **sum** | ***RvΔdKO***  **sum** | ***RvΔudgB***  **sum** | ***RvΔung*** | ***RvΔudgB*** | | ***RvΔdKO*** |
| A_G | 14 | NA | 4 | 0.795454545 | 0.227272727 | | NA |
| A_T | 4 | NA | 4 | 0.227272727 | 0.227272727 | | NA |
| C_A | 8 | 3 | 9 | 0.454545455 | 0.511363636 | | 0.227272727 |
| C_G | NA | 3 | 3 | NA | 0.170454545 | | 0.227272727 |
| G_A | 13 | 1 | 12 | 0.738636364 | 0.681818182 | | 0.075757576 |
| G_C | 8 | NA | 8 | 0.454545455 | 0.454545455 | | NA |
| T_C | 4 | 3 | 7 | 0.227272727 | 0.397727273 | | 0.227272727 |
| T_G | 12 | 1 | 12 | 0.681818182 | 0.681818182 | | 0.075757576 |
|  |  |  |  |  | |  |  |
