# Supplementary material for: Compromised base excision repair pathway in Mycobacterium tuberculosis imparts superior adaptability in the host
Source: PLoS Pathog. 2021 Mar 19;17(3):e1009452. doi: 10.1371/journal.ppat.1009452 (PMC8011731; doi:10.1371/journal.ppat.1009452)
Supplement: S6 Table — (DOCX) [file ppat.1009452.s016.docx]

**S6 Table. Mutation Spectrum of *Rv (GP).***

| **Table S6: Mutation Spectrum of *Rv* (GP)** | | | | | | | | | | | | | |
| --- | --- | --- | --- | --- | --- | --- | --- | --- | --- | --- | --- | --- | --- |
|  |  |  |  |  |  |  |  |  |  |  |  |  | **Mutation per million bp** |
| **Mutation** | ***Rv* (GP) 1** | ***Rv* (GP) 2** | ***Rv* (GP) 3** | ***Rv* (GP) 4** | ***Rv* (GP) 5** | ***Rv* (GP) 6** | ***Rv* (GP) 7** | ***Rv* (GP) 8** | ***Rv* (GP) 9** | ***Rv* (GP) 10** | ***Rv* (GP) 11** | **sum** | **(*Rv* GP)** |
| C_A | 1 | 1 | NA | 1 | NA | 1 | NA | NA | 1 | 1 | NA | 6 | 0.123966942 |
| C_G | 1 | NA | NA | 1 | NA | 1 | NA | NA | NA | 1 | NA | 4 | 0.082644628 |
| C_T | 1 | 1 | NA | 1 | 1 | 1 | NA | NA | 1 | 1 | NA | 7 | 0.144628099 |
| G_T | NA | NA | 1 | NA | NA | NA | NA | NA | NA | NA | 1 | 2 | 0.041322314 |
| T_G | NA | NA | 1 | NA | 1 | NA | NA | NA | 1 | NA | NA | 3 | 0.061983471 |
| G_A | NA | NA | NA | NA | 1 | NA | NA | NA | NA | NA | NA | 1 | 0.020661157 |
| A_G | NA | NA | NA | NA | NA | NA | 1 | 1 | NA | NA | NA | 2 | 0.041322314 |
| G_C | NA | NA | NA | NA | NA | NA | 1 | NA | NA | NA | NA | 1 | 0.020661157 |
