# Supplementary material for: Compromised base excision repair pathway in Mycobacterium tuberculosis imparts superior adaptability in the host
Source: PLoS Pathog. 2021 Mar 19;17(3):e1009452. doi: 10.1371/journal.ppat.1009452 (PMC8011731; doi:10.1371/journal.ppat.1009452)
Supplement: S8 Table — (DOCX) [file ppat.1009452.s018.docx]

| **Table S8: Mutation spectrum of *Rv* (GP)*, RvΔung* (GP)*, RvΔudgB* (GP) *and RvΔdKO* (GP)** | | | | | | | | |
| --- | --- | --- | --- | --- | --- | --- | --- | --- |
|  |  |  |  |  | **Mutation per million bp** | | | |
| **Mutation** | ***RvΔdKO* (GP) sum** | ***Rv* (GP) sum** | ***RvΔudgB* (GP)**  **sum** | ***RvΔung*(GP) sum** | ***Rv* (GP)** | ***RvΔung* (GP)** | ***RvΔudgB* (GP)** | ***RvΔdKO* (GP)** |
| A_G | NA | 2 | NA | NA | 0.041322314 | NA | NA | NA |
| C_A | 8 | 6 | 9 | 8 | 0.123966942 | 0.227272727 | 0.255681818 | 0.202020202 |
| C_G | 9 | 4 | 8 | NA | 0.082644628 | NA | 0.227272727 | 0.227272727 |
| C_T | 65 | 7 | 72 | 5 | 0.144628099 | 0.142045455 | 2.045454545 | 1.641414141 |
| G_A | 71 | 1 | 86 | NA | 0.020661157 | NA | 2.443181818 | 1.792929293 |
| G_C | NA | 1 | NA | NA | 0.020661157 | NA | NA | NA |
| G_T | NA | 2 | NA | NA | 0.041322314 | NA | NA | NA |
| T_C | 8 | NA | 8 | 1 | NA | 0.028409091 | 0.227272727 | 0.202020202 |
| T_G | 3 | 3 | 2 | 6 | 0.061983471 | 0.170454545 | 0.056818182 | 0.075757576 |

**S8 Table. Mutation spectrum of *Rv (GP), RvΔung (GP), RvΔudgB (GP)* and  *RvΔdKO(GP).***
