# Supplementary material for: Compromised base excision repair pathway in Mycobacterium tuberculosis imparts superior adaptability in the host
Source: PLoS Pathog. 2021 Mar 19;17(3):e1009452. doi: 10.1371/journal.ppat.1009452 (PMC8011731; doi:10.1371/journal.ppat.1009452)
Supplement: S10 Table — (DOCX) [file ppat.1009452.s020.docx]

**S10 Table. Mutation spectrum of *RvΔdKO in vitro* and *RvΔdKO* ciprofloxacin resistant strains.**

| **Table S10: Mutation spectrum of *RvΔdKO in vitro* and *RvΔdKO* CR** | | | | |
| --- | --- | --- | --- | --- |
|  |  |  | **Mutation per million bp** | |
| **Mutation** | ***RvΔdKO*** | ***RvΔdKO* CR** | ***RvΔdKO*** | ***RvΔdKO CR*** |
| A_G | NA | 13 | NA | 0.227272727 |
| C_A | NA | 1 | NA | 0.017482517 |
| C_T | NA | 63 | NA | 1.101398601 |
| G_A | 1 | 46 | 0.025252525 | 0.804195804 |
| T_G | 1 | 6 | 0.025252525 | 0.104895105 |
