# Supplementary material for: Compromised base excision repair pathway in Mycobacterium tuberculosis imparts superior adaptability in the host
Source: PLoS Pathog. 2021 Mar 19;17(3):e1009452. doi: 10.1371/journal.ppat.1009452 (PMC8011731; doi:10.1371/journal.ppat.1009452)
Supplement: S11 Table — (DOCX) [file ppat.1009452.s021.docx]

**S11 Table. List of DNA oligomers used in the study.**

| S.NO. | **Table 11: List of oligonucleiotides** | |
| --- | --- | --- |
| 1 | *rpoB* forward | 5’ CGACCACTTCGGCAACCG 3’ |
| 2 | *rpoB* reverse | 5’ CGATCAGACCGATGTTGG 3' |
| 3 | 5' flank forward primer of *ung* | 5*'* AAGCTTCGAGTCGCCGCATCCTCC 3*'* |
| 4 | 5' flank reverse primer of *ung* | 5*'* ACTAGTCGCAATACCGCGCCAGAG 3*'* |
| 5 | 3' flank forward primer of *ung* | 5' GGTACCGCTGGTGACAGATAGTCA 3*'* |
| 6 | 3' flank reverse primer of *ung* | 5*'* TCTAGAGCGCAACACATTCGATCC 3*'* |
| 7 | *Ung* knockout screening forward primer | 5’ CACTCACTTGCAACGGAGTCC 3’ |
| 8 | *Ung* knockout screening reverse primer | 5’ CAGCACCGAGACGATAGTTCC 3' |
| 9 | 5' flank forward primer of *udgB* | 5' TTTTTTTTCCACAAAGTGGTGATGCCGCCAACCG 3' |
| 10 | 5' flank reverse primer of *udgB* | 5' TTTTTTTTCCATTTCTTGGTTACGCCCGGCACACGCTGATC 3' |
| 11 | 3' flank forward primer of *udgB* | 5' TTTTTTTTCCACAGAGTGGGATTGAGTGACGTGAAGAC 3' |
| 12 | 3' flank reverse primer of *udgB* | 5' TTTTTTTTCCACCTTGTGGGTAGTCGTCACCGGCCGCC 3' |
| 13 | *UdgB* knockout screening forward primer | 5’ GATACAGCGCGGTGACGGTC 3’ |
| 14 | *UdgB* knockout screening reverse primer | 5’ ATGCACGATTCGTTCGAACTC 3’ |
| 15 | GU9 oligonuclieotide | 5'CTCAAGTGUAGGCATGCTTTTGCATGCCTGCACTTGA 3' |
| 16 | SSU9 oligonuclieotide | 5'CTCAAGTGUAGGCATGCAAGAGCT 3' |
| 17 | *Ung* gene forward primer with NdeI | 5’CACCCATATGGCCGCACGGCCGTTGAGT 3’ |
| 18 | *Ung* gene reverse primer with HindIII | 5’GATCAAGCTTCAGGGCAACCGCCAATCGAT 3’ |
| 19 | *UdgB* gene forward primer with NdeI | 5’cacccatatgaatatcgcggctgaatc 3’ |
| 20 | *UdgB* gene reverse primer with HindIII | 5’AGCTAAGCTTACTCAATCCCGGCCAGCTTC 3’ |
