# Supplementary material for: Compromised base excision repair pathway in Mycobacterium tuberculosis imparts superior adaptability in the host
Source: PLoS Pathog. 2021 Mar 19;17(3):e1009452. doi: 10.1371/journal.ppat.1009452 (PMC8011731; doi:10.1371/journal.ppat.1009452)
Supplement: Attachment — Submitted filename: Response_Commments.pdf [file ppat.1009452.s023.pdf]

Part I – Summary

Reviewer #1:

The goal of the authors of this paper set forth to establish the identity and functionality of annotated genes udg and ung as uracil glycosylases as well as to examine their role in DNA repair, mutagenesis and virulence in Mtb. The role of DNA repair systems in Mtb pathogenesis and mutagenesis is an important topic that is understudied. To pursue their goals, the authors create null mutants of TB uracil DNA glycosylases (ung and udgB) and construct complemented strains. The phenotypes of these mutants shown are:

- loss of uracil glycosylase activity in lysates.
- No growth defect for mutants under physiological conditions.
- Increase of the mutation rate in mutants under physiological conditions by RifR assay or ciprofloxacin resistance
- The ung deletion increases the mutation frequency during nitrosative stress/ the udgB deletion increases mutation frequency during oxidative stress.
- The deletion of both the genes confers hypervirulence in guinea pig infection as measured by granulomas and bacterial load.
- By WGS, colonies of ΔudgB and double mutant showed a higher number of SNPs compared with Δung and WT.
- The double mutant is more fit in serial passage and competition experiments in macrophage and mouse infection.

Overall, the topic is of interest and a new DNA repair pathway important for Mtb pathogenesis is an important finding. However, some problems with data presentations, controls, and interpretation somewhat limit the potential impact of the findings, as presented.

Reviewer #2:

The modulation of genomic integrity in *Mycobacterium tuberculosis* through spontaneous or stress-induced mutagenesis has been hypothesized to be central to the development of drug resistance in this organism. Consequently, the study of DNA repair pathways including nucleotide/base excision repair pathways, together with the activity of mutator polymerases, is expected to provide useful insight into the molecular mechanisms of drug resistance in mycobacteria. In this submission, Naz and colleagues assess the role of base excision repair in mutagenesis and the emergence of drug resistance in *M. tuberculosis*. The authors generate single and double mutants that lack the Udg and Ung uracil DNA glycosylases (UDGs) and test these in several model systems. The authors confirm that mutation of the corresponding genes results in loss of uracil DNA glycosylase activity in cell free extracts, genetic complementation restored the activity. The mutants did not display any growth defects under standard conditions in broth culture or stress conditions. Whole genome sequencing suggested that loss of these enzymes did not yield increased chromosomal mutations but was associated with increased mutation rate to Rifampin as determined by the Luria-Delbruck fluctuation assay. The mutants also appeared to have increased DNA damage-induced mutation frequencies in the presence of oxidative and nitrosative stress. In the guinea model of tuberculosis infection, the mutants displayed enhanced colonization of lung tissue when compared to the wild type and accumulated more mutations than the wild type. Upon treatment with ciprofloxacin, loss of UDGs was associated with accumulation of mutations in drug targets such as GyrA. In

competition experiments, the UDG double mutant out competed the wild type strain. The authors conclude the UDGs are important mediators of fitness/adaptation and drug resistance. Overall, there are some interesting data here but there are also notable concerns.

Reviewer #3:
The manuscript by Naz et al describes the characterization of uracil glycosylases mutant strains ung and udgB with regards to growth profiles and mutation spectrum analyses, both in vitro and in vivo. The authors generate deletion strains of ung, udgB, and the double mutant, along with complemented strains for each. They found that the single mutants had slight increases in mutation rates, while the double mutant was up 22-fold (using spontaneous Rif resistance analysis). They also looked at RifR mutation rate in response to nitrositive and oxidative stresses, with differential increased mutation rates among the single and double mutants.

The authors go on to use the guinea pig model of infection to assess the survival and mutability of their uracil glycosylase mutants in vivo. They found that the double mutant (RvΔdKO) showed a ~0.5 log increase in CFU's (after 56 days), suggesting this strain is more virulent compared to wild type Rv. Using WGS analysis, colonies derived from post-infection of the mutant strains showed higher rates of SNP accumulation for the udgB and RvΔdKO strains, suggesting a link between the hyper virulence of RvΔdKO and the hypermutability of the uracil glycosylase mutants in vivo. Finally, the authors go on to show that the loss of UDG's accelerated evolution to ciprofloxacin resistance, and that the RvΔdKO has a competitive advantage over Rv in peritoneal macrophages, but only after successive rounds of infection.

Overall, the paper is well written and describes a thorough series of experiments showing how excision repair mutants, incapable of excising uracil from DNA, generate SNPs under stress *in vivo*. This mutability ultimately gives them a path that allows them to escape host defenses, leading to increased virulence and competitive growth advantages. While such studies have been performed in other pathogens (as discussed by the authors in th Discussion), I have not seen such a thorough analysis of the evolvability of DNA repair mutants in *M. tuberculosis* before.

Response.
We thank the editor and reviewers for their insightful comments. We also want to thank the reviewers for encouraging comments about the work. We have made earnest efforts to address all the questions raised by reviewers by performing all the suggested experiments. We believe that in addressing the questions, both the quality and presentation of the manuscript have improved.

Four main questions:

1. Comment.

One question that is unclear to me, and perhaps the authors could help with this. When they lists the results of mutation spectrums from GWS analyses, are the base changes listed in the figures in tables (e.g., C > T) for one strand of the chromosome? Otherwise, wouldn't the number of C > T changes always equal the number G > A changes in a genome wide analysis? In other words, what distinguishes a C > T change from a G > A change in Fig. 5e for any CG base pair. A clarification would help those us who don't see mutation spectrum analyses often enough. An explanation on the above question may clarify a related concern regarding the mutation spectrum analysis discussed in the text and shown in Figure 4h.

Response.

RvΔung, RvΔudgB, and RvΔdKO were generated in laboratory Rv background (Rv in vitro); therefore, we have used Rv in vitro as the reference genome for the analysis. Mutations listed are indeed for one strand of DNA. This fact has been clearly mentioned in the figure legends. The mutation spectrum analysis was performed by counting the number of independent mutations per gene; therefore, C→T mutation is different from G→A mutation.

The authors state in lines 205-206 that "A spectrum analysis of mutations showed a clear predisposition towards G>A and C>T mutations…:., but the data show that the increase in mutations in the udgB and RvΔdKO strains are mostly T>C and C>A, mutations not to be expected from loss of uracil glycosylase.

Response:

We apologize for the labeling error. We have corrected the error in the revised version of the manuscript.

2. Comment.

Can the authors speculate as to why with a 0.5-fold increase in CFUs with the RvΔdKO stain (Fig. 3c), they see a lower rate of granuloma formation of RvΔdKO relative to the udgB strain?

Response:

Thank you for the comment. We had simply counted the number of granulomas visible on the surface in the captured images. Since the data depends on the image captured, we have removed it from the revised manuscript. In the revised manuscript, we reperformed the guinea pig infection experiment with Rv, RvΔdKO, and the complementation strain RvΔdKO::ung-udgB. The hypervirulence phenotype observed was reproducible (Fig 4h), and importantly, complementation strain restored the phenotype. We have performed Hematoxylin and eosin staining to examine the gross histopathology. Results showed the presence of well-formed granulomas in all three strains (Fig. 4i).

3. Comment.

If I understand Fig. 3b correctly, the heat map shows the percentage of colonies picked for any one mutant that contains that SNP. Perhaps unexpectantly, the results show that many of the SNPs are present at 100% in the single mutants (i.e., 4 out of 4), but are totally absent in the double mutant (0%). Is this counterintuitive?

Response:

We agree with the reviewer. The result was indeed counterintuitive. The majority of the SNPs were found in PPE and PE_PGRS family genes (Fig. 3b). Collectively out of the 24 SNPs, 12 were present in PE_PGRS5, 8 mutations in PPE60, and 2 mutations in PPE47. PE and PPE family of proteins in Mtb represent ~10% of its coding regions. Due to the presence of repetitive sequences and high GC rich content, these regions cannot be sequenced and aligned with high confidence.

4. Could the authors speculate what other repair genes, if inactivated, might recapitulate their results with the UNG deletion strain. I gather it would include mutations in repair genes that include a low to moderate increase in mutation rate (like ~ 20%). Included in this discussion would be the acknowledgement that some mutants would be too mutagenic to allow evolution to occur (e.g., a proofreading mutation in dnaE1, or the recently described endoMS/nucS mutation in mismatch repair).

Response:

Thank you for the comment. We agree with the reviewer and discussion has been modified to include these points.

"DNA repair genes such as UDGs or other BER pathway genes, upon deletion, exhibit approximately 20 % higher mutation rate, which would help in bacillary survival (1,2). How is such a property advantageous to the pathogen under physiological conditions? Many DNA repair enzymes, including ung and udgB are downregulated during hypoxia (3). This may explain the broad mutation spectrum observed in the strains isolated from the infected guinea pig lung granulomas, an oxygen-depleted environment (Fig 6f). An advantage of such a regulation could be that when Mtb reactivates from dormancy, it carries a repertoire of population that offers better fitness to the bacterium to sustain itself for active growth in the changed physiological environment. The bacterium that reactivate from dormancy may also offer potential advantages in evolving/acquiring drug resistance. In contrast, deletion of genes such as dnaE1 or endoMS/nucS results in a very high mutation rate that eventually compromises the strain's fitness (4,5)"

Part II – Major Issues: Key Experiments Required for Acceptance

Reviewer #1:

Comment:

The finding of a hypervirulent phenotype in a mutant is exciting but should be interpreted cautiously. There are examples of such findings being due to spontaneous attenuating mutations in the wild type stock, which do not carry over to the mutant because of clonal selection in the construction of the mutant. This gives the appearance of hypervirulence. This issue is solved by in vivo testing of a complemented strain, which should reverse the hypervirulence.

Response:

We agree with the observation of the reviewer. To negate this possibility, we reperformed the guinea pig infection experiment with Rv, RvΔdKO, and the complementation strain RvΔdKO::ung-udgB. Implantation of Rv, RvΔdKO and RvΔdKO::ung-udgB at day 1 p.i in guinea pigs' lungs was comparable (Figs 4g-h). While the CFUs obtained 56-days p.i were similar for Rv, and RvΔdKO::ung-udgB, we noticed an ~0.5 log fold increase in the CFUs of RvΔdKO in lungs and spleen (Figs. 4h). Hematoxylin and eosin staining performed to examine the gross histopathology showed the presence of well-formed granulomas in all three strains (Fig. 4i). The results are consistent with the earlier guinea pig infection experiment (Fig S4b). The complementation strain's ability to restore the phenotype suggested that RvΔdKO's hypervirulence is not due to the spontaneous reversion of attenuating mutations.

Comment:

A major conclusion of the paper is that accumulation of mutations in UDG mutants confers fitness during infection due to enhanced evolutionary traits, but this interpretation is difficult to prove. For example, it could be that uracil excision is lethal in the setting of high uracil load and therefore the absence of the repair system provides an advantage.

Response:

We thank the reviewer for the comment. The phenomenon is not limited to UDG mutants. Mtb genes involved in NHEJ, HR, and BER pathways were found to be dispensable for in vitro growth as well as in vivo survival. Deletions of genes of the NHEJ and HR double-stranded DNA repair pathways independently or together did not compromise Mtb survival in different animal infection models (6). Deletion of AP-endonucleases, endA, and xthA, independently or together had no impact on mycobacterial survival in a guinea pig model of infection (7). DNA repair genes such as UDGs or other BER pathway genes, upon deletion, exhibit approximately 20 % higher mutation rate, which would help in bacillary survival (1,2) WGS analysis of RvΔdKO showed that genes such as vapC47, gadB, eccD4, PE-PGRS9, embB, and fadD4 possess non-synonymous mutations (Figs 4d; S4d). Deletion of any of these genes provides a growth advantage to the pathogen (8). Interestingly, a non-sense mutation in the Rv3437 at Glu-69 position was identified in RvΔdKO. Disruption of this gene also provides a growth advantage to the bacteria (https://mycobrowser.epfl.ch/) (Fig. S4d).

Comment:
The assertion that these strains can be used to screen for targets of drugs isn't convincing and doesn't add to the story that is presented (ie much of Figure 5). The mutations are not functionally verified and cannot be directly linked to drug action. This line of investigation would be best excluded from the paper to instead focus on the other phenotypes of the mutants.

Response:

Whole genome sequence analysis of ciprofloxacin resistant RvΔdKO identified mutations in gyrA, nanT, and ureC, in multiple ciprofloxacin-resistant strains (≥40%). As expected, the analysis showed mutations in gyrA, a direct target of fluoroquinolones. A→ G or G→A mutation in gyrA that results in Asp→Asn or Gly at the 94th position occur in all fluoroquinolone-resistant isolates (39). Since the identified mutations are already linked to ciprofloxacin resistance (39), we have not performed functional validation experiments.

Reviewer #2:
1. Comment.
Mutation frequency experiments in figure 3 F&G appear to be done on select strains for select assays. Why are all strains not shown on this graph? In addition, genetically complemented strains are missing . Why is this the case?

Response:

We included complementation strains and repeated the mutation frequency experiment in the presence of acidified sodium nitrite (source of RNI) and cumene hydroperoxide (CHP) (source of ROS) (Fig 3f). The complementation strains restored the phenotype in both the stress conditions. These results suggest the differential abilities of Ung and UdgB in repairing DNA damage in the host.

2. Comment.
For the guinea pig infections, it is unclear how the data on numbers of granulomas was determined. There is not sufficient detail in the methods section nor the figure legend to determine this. It seems difficult to count granulomas by just looking at the surface of excised lungs. This would require sectioning and some form of morphometry on serial sections. Moreover, the gross morphology shown in Figure 4A is unconvincing. It is not immediately apparent that there is a difference between the wild type and the mutants. Furthermore, data on the genetically complemented strains is not presented for any of the animal data. This significantly weakens the study

Response.

Thank you for the comment. We had simply counted the number of granulomas visible on the surface in the captured images. Since the data depends on the image captured, we have removed it from the revised manuscript. In the revised manuscript, we reperformed the guinea pig infection experiment with Rv, RvΔdKO, and the complementation strain RvΔdKO::ung-udgB. Implantation of Rv, RvΔdKO and RvΔdKO::ung-udgB at day 1 p.i in guinea pigs' lungs was comparable (Figs 4g-h). While the CFUs obtained 56-days p.i were similar for Rv, and

RvΔdKO::ung-udgB, we noticed an ~0.5 log fold increase in the CFUs of RvΔdKO in lungs and spleen (Figs. 4h). Thus hypervirulence phenotype observed was reproducible, and importantly, complementation strain restored the phenotype. We have performed Hematoxylin and eosin staining to examine the gross histopathology. Results showed the presence of well-formed granulomas in all three strains (Fig. 4i).

3. Comment.
What is the purpose of showing the genes wherein mutations accumulated during guinea infection? It seems that the more interesting data here would be the nature of the mutation and if there were any hotpots in drug resistance conferring loci. The rest of the information is not useful.

Response.
We concur with the views expressed by the reviewer. Data suggests that RvΔdKO exhibit better survival in the guinea pigs and accumulated more mutations; therefore, we sorted the data based on the nature of mutations. The analysis showed that 34% of mutations are synonymous, 58% mutations are non-synonymous, 6.4% are intergenic mutations, and 1.6% have non-sense mutations. Further analysis showed that genes such as vapC47, gadB, eccD4, PE-PGRS9, embB, and fadD4 possess non-synonymous mutations. Deletion of any of these genes provides a growth advantage to the pathogen. Interestingly, a non-sense mutation in the Rv3437 at Glu-69 position was identified in RvΔdKO. Disruption of this gene also provides a growth advantage to the bacteria (https://mycobrowser.epfl.ch/) (Fig. S4d). However, we did not identify mutations in the drug resistance-conferring loci such as inhA, rpoB, or katG. This is most likely because guinea pigs were not subjected to antibiotics treatment before or after the infection.

4. Why was ciprofloxacin used to determine if UDG deletion can accelerate mutation rate? The choice of this drug needs to be rationalized as treatment with ciprofloxacin would have overlapping effects with loss of UDGs in the mutant due to the induction of a DNA-damage response and the possible induction of the activity of mutator polymerases. Perhaps this point is illustrated by the very high mutation rates reported for the fluoroquinolone used. Using a drug that induces a DNA damage response does not make intuitive sense when trying to determine the mutator effect of UDG loss. This should be attempted with another drug that does not affect DNA metabolism.
Response:
We agree with the reviewer that ciprofloxacin and rifampicin can accelerate mutation rates since they are involved in DNA metabolism/transcription. As suggested, we have performed mutation rate analysis using isoniazid, which does not impact DNA metabolism. We observed a 5.32, 22.11, and 37.17-fold increase in the spontaneous mutation rate of RvΔung, RvΔudgB, and RvΔdKO strains compared with Rv in the presence of isoniazid (Figs. 3d-e).

5. It is unclear what the authors are trying to illustrate with the competition experiment as the result is not sufficiently resolved. The difference between the double mutant and wild type is convincing but why does this happen in competition and not in individual culture? What is the mechanism?

Response:

If a strain has superior fitness over the other, one would expect that it would show relatively higher CFUs with time. Even a weaker strain can continue to grow in a given environment in the absence of a competing fitter strain. The difference would only be apparent when both the strains compete for the same niche. To decisively determine if RvΔdKO is indeed a fitter strain compared with Rv, we performed a competition experiment in guinea pigs (Fig 6e & 7a). We competed Rv with RvΔdKO. Besides, we also competed Rv with complemented strain RvΔdKO::ung-udgB. This experiment is part of the revised manuscript.

"CFU plating at 56 days p.i suggests ~0.5 to 1 log fold increase in the survival of RvΔdKO in comparison with Rv in lungs and spleen, respectively (Fig. 6e). In case of Rv and RvΔdKO::ung-udgB, no apparent difference was observed (Fig. 6e). Percent CFUs analysis showed an equal deposition of strains at day 1 in mice and in guinea pigs lungs. Survival difference between Rv and RvΔdKO became evident at day 56 p.i in the lungs and spleen (Fig. 7a). Together, these results suggest that RvΔdKO displays a decisive edge over the wild-type parent (Fig. 7b). We speculate that this is due to the strain's ability to develop mutations that provide a survival advantage. Collectively, deletion of BER genes ung and udgB drives the accumulation of mutations in the genome under stress conditions and assists in enhanced adaptation."

Reviewer #3: No additional experiments required.

Part III – Minor Issues: Editorial and Data Presentation Modifications

Please use this section for editorial suggestions as well as relatively minor modifications of existing data that would enhance clarity.

Reviewer #1: 1) The Supplementary figures are missing from the SI file (only legends and tables are present).

Response: We apologize for the oversight. We seemed to have missed out submitting supplementary figures while uploading.

2) Although the verification of the mutant genotypes is convincing and done by multiple methods, some of the PCR verification could be improved (for example 1B, F2-R2 WT is not clear).

Response. We made efforts to improve this image. Unfortunately, we continue to get the background.

3) In Figure 2b, why is the inoculum in Sauton's medium so high?

Response.

The experiment has been repeated with lower inoculum (Fig 2b).

4) Figure 2e: a part of the glycosylase activity is due to ung (decreased product formation in the ung mutant) but there is no activity at all in the udgB single mutant (where is the ung-dependent activity?): how do the authors explain this result?

Response:

We apologize for the labeling mistake. The experiment was performed in the presence of Ugi (Fig 2e).

5) The mutation spectrum in mutant vs WT in Rif experiments is not reported. They also should regroup the data so that the same mutations are together (e.g.G>A=C>T)

Response: We apologize for the oversight. We seemed to have missed out on submitting supplementary figures while uploading. The mutation spectrum is reported in Fig S3.

6) How do the authors explain that there is no increase in spontaneous mutations observed using WGS but they detect an increase with rifR and CipR resistance as a readout?

Response:

Results show that the deletion of UDGs did not result in the accumulation of significant mutations when cultured in a nonselective complete medium. Suggesting that in the absence of external stress, the presence or absence of a particular repair gene may be of little consequence as the alternate DNA repair pathways may substitute for the deficiency. However, strains subjected to nitrosative or oxidative, or antibiotic stress, repair mutants displayed hypermutability (Figs. 3d-f).

7) The text states that there is a "Predisposition towards G>A and C>T mutations" (Figure 4h: lines 205-208), but the data in the figure does not support this, those mutation types are no more common than C>A or T>C.

Response.
We apologize for the labeling error. We have corrected the error in the revised version of the manuscript.

8) Competition experiments: in macrophages (figure 6c) is accomplished by screening based on HygR/S. Is it possible some of the HygR colonies are due to chromosomal mutagenesis rather than present of the HygR marker given the mutagenic phenotypes of the strains?
Response.
We agree that such a possibility certainly exists. However, we do not believe that to be the case in our experiment as the difference could only be observed wild type was competed against double KO, not when wild type competed against complemented strain.

9) In the mouse experiments (Figure 6e): the data is difficult to understand and the phenotype is weak. What is the Y axis here? This data should present the result as in Figure 6c (proportion of each strain in the mix).
Response.
As suggested, we have represented as percent CFUs in Fig 7a.

Optimally, this result should also include a control of WT-hyg/WT-kan competition to control for differences in plating efficiency between the markers.

Response.
We have reperformed the competition experiment in guinea pigs (Fig 6e & 7a). We competed Rv with RvΔdKO. Besides, we also competed Rv with complemented strain RvΔdKO::ung-udgB. This experiment is part of the revised manuscript.

Other Comments:
1) The text states "2 biological replicates in triplicate" Were statistical tests done with n=6 or n=2?
Response.
While each experiment was performed minimally biologically twice, results show the data from one of the biological experiments performed in technical triplicates. Therefore n=3.

2) Fig 2d and e are not properly labeled to differentiate the samples incubated with/ without Ugi. udgB is complemented? Is the protein being under expressed?

Response.
There was a labeling error, which has now been corrected. While we realize that the activity is not precisely equivalent to complementation (Fig 2e), the complementation strains work very well in mutation frequency and ex vivo, in vivo experiments.

Δ3) 2e) Is there a reason for the low level of product when
Response.
While we realize that the activity is not precisely equivalent to complementation (Fig 2e), the complementation strains work very well in mutation frequency and ex vivo, in vivo experiments.

4) "Next we analyzed the 159 unique SNPs that were found in RvΔung, RvΔudgB, and RvΔdKO in comparison with Rv in vitro (Figs. 3c)" Should be 3b.
Response.
We apologize for the error. We have corrected the error in the revised version of the manuscript.

5) "To examine the acquisition of spontaneous mutations in the absence of ung, udgB, or both, we performed mutation rate analysis using a fluctuation test (Figs. 2c-d)" This refers to 3c.
Response.

We apologize for the error. We have corrected the error in the revised version of the manuscript.

6) "Collectively the data suggests that UDG mutants were hypermutable (Figs. 2d-e)" – Also think this is wrong. Should be 3d-e

Response.

We apologize for the error. We have corrected the error in the revised version of the manuscript.

7) 6b and 6C: The number of replicates used is not given.

Response. Modified the legend to include the data on replicates.

8) Figure 6e does not have Y axis label.

Response.

We apologize for the labeling error. We have corrected the error in the revised version of the manuscript.

9) What is the statistical test used in all panels? It should be given in each legend or explained in a specific paragraph in material and method (only given for figure 3d).

Response.

Statistical test used has been clearly mentioned in the legends.

10) The global organization of the paper could be improved. We suggest:

a. Spontaneous mutation (WGS, rif, cip)

b. Stress- and infection- induced mutation

c. Hyper infection phenotypes (macrophage, mice, guinea pig)

Response.

Modified as per suggestions.

Reviewer #2: Figure 1A-E: It is unclear what the authors intend to show here. Without the sizes of the expected PCR product, or the sizes of the markers, the illustrations and gels are not useful to determine the genetic integrity of the strains used in the study. Whilst some sizes are indicated in the legend it is difficult to follow. Furthermore, Southern Blots are the more acceptable way of confirming genetic mutants.

Response: We thank the reviewer for pointing it out. Figure 1B,1D, and 1E are labeled properly in the revised manuscript. We agree that southern blots would be a terrific way to confirm the KOs. However, we have confirmed the KOs by western blots and activity assays.

Figure 4C is referred to in the text prior to Figure 4A and B.
Response.
We apologize for the error. We have corrected the error in the revised version of the manuscript.

Figure 5 A should be removed and given as MIC values in the text.
Response: As suggested, we have moved the figure to supplementary (Fig S4e).

Reviewer #3: Minor points:

1. Authors should report the concentrations of antibiotics used in the study.
Response:
Thank you for pointing out. We have reported the concentrations of antibiotics in the revised manuscript.

2. Line 159-160. Figure cited should be Fig. 3b, not 3c.
Response.
We have corrected the error in the revised version of the manuscript.

3. Lines 167 and 173. Figures cited should be Fig. 3c-d, not Fig. 2 c-d.
Response.
We have corrected the error in the revised version of the manuscript.

4. Line 200: Since the authors are discussing the vivo results, I think they mean Fig. 4 (e-f).
Response.
Thank you for pointing it out. We have corrected the error in the revised version of the manuscript.

5. Line 202: .....showed significant SNP accumulation in UDG mutant strains.
Authors should note the figure that shows this (i.e, Fig. 4g).
Response.
We have corrected the error in the revised version of the manuscript.

6. Line 232: The authors should use the 3 letter codes for amino acids here (Asp>Asn or Gly), to prevent confusion when talking about A>G or G>A base pair changes in the same sentence.
Response: Thank you. We have incorporated the changes in the revised manuscript.

References

1. Kurthkoti, K., Srinath, T., Kumar, P., Malshetty, V.S., Sang, P.B., Jain, R., Manjunath, R. and Varshney, U. (2010) A distinct physiological role of MutY in mutation prevention in mycobacteria. *Microbiology*, **156**, 88-93.
2. Hassim, F., Papadopoulos, A.O., Kana, B.D. and Gordhan, B.G. (2015) A combinatorial role for MutY and Fpg DNA glycosylases in mutation avoidance in Mycobacterium smegmatis. *Mutat Res*, **779**, 24-32.
3. Kurthkoti, K. and Varshney, U. (2010) Detrimental effects of hypoxia-specific expression of uracil DNA glycosylase (Ung) in Mycobacterium smegmatis. *J Bacteriol*, **192**, 6439-6446.
4. Castaneda-Garcia, A., Prieto, A.I., Rodriguez-Beltran, J., Alonso, N., Cantillon, D., Costas, C., Perez-Lago, L., Zegeye, E.D., Herranz, M., Plocinski, P. *et al.* (2017) A non-canonical mismatch repair pathway in prokaryotes. *Nat Commun*, **8**, 14246.
5. Rock, J.M., Lang, U.F., Chase, M.R., Ford, C.B., Gerrick, E.R., Gawande, R., Coscolla, M., Gagneux, S., Fortune, S.M. and Lamers, M.H. (2015) DNA replication fidelity in Mycobacterium tuberculosis is mediated by an ancestral prokaryotic proofreader. *Nat Genet*, **47**, 677-681.
6. Heaton, B.E., Barkan, D., Bongiorno, P., Karakousis, P.C. and Glickman, M.S. (2014) Deficiency of double-strand DNA break repair does not impair Mycobacterium tuberculosis virulence in multiple animal models of infection. *Infect Immun*, **82**, 3177-3185.
7. Puri, R.V., Reddy, P.V. and Tyagi, A.K. (2014) Apurinic/apyrimidinic endonucleases of Mycobacterium tuberculosis protect against DNA damage but are dispensable for the growth of the pathogen in guinea pigs. *PLoS One*, **9**, e92035.
8. DeJesus, M.A., Gerrick, E.R., Xu, W., Park, S.W., Long, J.E., Boutte, C.C., Rubin, E.J., Schnappinger, D., Ehrt, S., Fortune, S.M. *et al.* (2017) Comprehensive Essentiality Analysis of the Mycobacterium tuberculosis Genome via Saturating Transposon Mutagenesis. *mBio*, **8**.
